# Analysis of cancer-associated fibroblasts related genes identifies COL11A1 associated with lung adenocarcinoma prognosis

**DOI:** 10.1186/s12920-024-01863-1

**Published:** 2024-04-22

**Authors:** Haosheng Zheng, Jian Tan, Fei Qin, Yuzhen Zheng, Xingping Yang, Xianyu Qin, Hongying Liao

**Affiliations:** 1 Department of Thoracic Surgery, Thoracic Cancer Center, The Sixth Affiliated Hospital, Sun Yat-sen University, Guangzhou, Guangdong China; 2 Biomedical Innovation Center, The Sixth Affiliated Hospital, Sun Yat-sen University, Guangzhou, Guangdong China

**Keywords:** COL11A1, Lung adenocarcinoma, Immune checkpoint genes, WGCNA, Prognosis, Biomarker

## Abstract

**Background:**

The treatment of lung adenocarcinoma is difficult due to the limited therapeutic options. Cancer-associated fibroblasts play an important role in the development of cancers. This study aimed to identify a promising molecular target associated with cancer-associated fibroblasts for the treatment of lung adenocarcinoma.

**Methods:**

The Cancer Genome Atlas lung adenocarcinoma dataset was used to screen hub genes associated with cancer-associated fibroblasts via the EPIC algorithm and Weighted Gene Co-expression Network Analysis. Multiple databases were used together with our data to verify the differential expression and survival of COL11A1. Functional enrichment analysis and the single-cell TISCH database were used to elucidate the mechanisms underlying COL11A1 expression. The correlation between COL11A1 and immune checkpoint genes in human cancers was also evaluated.

**Results:**

Using the EPIC algorithm and Weighted Gene Co-expression Network Analysis, 13 hub genes associated with cancer-associated fibroblasts in lung adenocarcinoma were screened. Using the GEPIA database, Kaplan-Meier Plotter database, GSE72094, GSE75037, GSE32863, and our immunohistochemistry experiment data, we confirmed that COL11A1 overexpresses in lung adenocarcinoma and that high expression of COL11A1 is associated with a poor prognosis. COL11A1 has a genetic alteration frequency of 22% in patients with lung adenocarcinoma. COL11A1 is involved in the extracellular matrix activities of lung adenocarcinoma. Using the TISCH database, we found that COL11A1 is mainly expressed by cancer-associated fibroblasts in the tumor microenvironment rather than by lung adenocarcinoma cells. Finally, we found that COL11A1 is positively correlated with HAVCR2(TIM3), CD274 (PD-L1), CTLA4, and LAG3 in lung adenocarcinoma.

**Conclusion:**

COL11A1 may be expressed and secreted by cancer-associated fibroblasts, and a high expression of COL11A1 may result in T cell exhaustion in the tumor microenvironment of lung adenocarcinoma. COL11A1 may serve as an attractive biomarker to provide new insights into cancer therapeutics.

**Supplementary Information:**

The online version contains supplementary material available at 10.1186/s12920-024-01863-1.

## Introduction

Lung cancer is one of the leading causes of death in both men and women worldwide [1]. Lung adenocarcinoma (LUAD) is the most common histological subtype of lung cancer [2]. The prognosis of LUAD has greatly improved owing to the development of various LUAD therapies, such as surgery, chemotherapy, radiotherapy, targeted therapy, and immunotherapy [3]. However, many patients with LUAD are diagnosed in the advanced stages. Hence, the 5-year overall survival rate is low [4]. Therefore, there is an urgent need to identify effective biomarkers for the prevention and treatment of LUAD.

The development of tumors is affected not only by cancer cells but also by the tumor microenvironment (TME) [5]. TME refers to the tumor ecosystem within the human body consisting of immune cells, extracellular matrix, blood vessels, and other cells, such as fibroblasts. Tumor cells constantly interact within the microenvironment, both positively and negatively. Within the TME, the tumor stroma critically impacts the tumor’s invasiveness by regulating extracellular matrix depositions and cancer cell metabolism [6]. In recent years, cancer-associated fibroblasts (CAFs) and tumor stromal cells have received extensive attention. Some studies have indicated that CAFs are one of the most abundant stromal cell components in the TME and play an important role in tumor invasion, angiogenesis, and extracellular matrix remodeling by promoting cell-cell interactions and secretion of pro-invasive factors [7–9]. As such, CAFs are important for the discovery of therapeutic biomarkers for LUAD.

In this study, we used the immune infiltration score EPIC algorithm (http://epic.gfellerlab.org) [10] and Weighted Gene Co-expression Network Analysis (WGCNA) [11] methods to screen hub genes associated with CAFs in The Cancer Genome Atlas (TCGA) LUAD dataset. Hub genes were further validated using relevant datasets, and COL11A1 was identified as a potential biomarker for the prognosis and treatment of LUAD.

## Materials and methods

### Data collection and processing

Clinical information and RNA sequencing data were downloaded from The TCGA (https://portal.gdc.cancer.gov/) and Gene Expression Omnibus (GEO) databases (https://www.ncbi.nlm.nih.gov/geo/). The inclusion criteria were as follows: (a) samples diagnosed with LUAD; (b) number of samples more than 100; (c) cancer samples with clinical information including sample serial number, survival time, and survival status; (d) if the dataset lacked clinical data, the data is included unless the dataset contained paired samples (tumor vs. normal). The exclusion criteria were as follows: (a) lung cancer samples without adenocarcinoma, (b) cancer samples without essential clinical information, and (c) samples with no expression value for over half of the genes. The basic information of the included datasets is presented in Table 1. The “biobase” package was used to normalize the data. The probes were labeled with gene symbols according to the annotation information on the platform. The average value was calculated when multiple probes corresponded to one gene. When one probe corresponded to multiple genes, the probe was eliminated. Then, a gene expression matrix was obtained.

**Table 1:** The basic information of TCGA and GEO datasets in the study

Dataset	Data type	Platform	Sample type		Clinical information
			Tumor	Normal	
TCGA	mRNA	Illumina HiSeq	516	58	yes
GSE75037	mRNA	Illumina HumanWG-6 v3.0 expression beadchip	83	83	no
GSE72094	mRNA	Rosetta/Merck Human RSTA Custom Affymetrix 2.0 microarray	442	0	yes
GSE32863	mRNA	Illumina HumanWG-6 v3.0 expression beadchip	58	58	no

### Differentially expressed genes (DEGs) and immune infiltration analysis

The “Limma” package in R was used to screen DEGs between cancer and normal tissues in the TCGA LUAD dataset (|log2FC|> 1, FDR < 0.05). The " ggplot2” package in R was used to draw a volcano plot and heatmap (including the top 50 upregulated and top 50 downregulated genes) of DEGs. Next, we scored each sample in the TCGA LUAD dataset for immune infiltration using the EPIC algorithm (http://epic.gfellerlab.org). Subsequently, a variance analysis of immune infiltration scores between the tumor and normal groups was performed.

### Weighted gene co-expression network construction

The R package WGCNA was used to identify CAF-related modules and hub genes among DEGs. First, the outlier samples were removed by the “good-samples-gene” function in WGCNA. The adjacency matrix was transformed into a topological overlap matrix (TOM). Genes were divided into different modules according to the TOM-based dissimilarity measure. We set the soft-thresholding power to 8 (scale-free R^2^ = 0.88, networkType=“unsigned”), cut-off to 0.25, deepSplit to 3, and the minimal module size to 30 to identify key modules. The module with the highest correlation with CAFs was selected for subsequent analyses.

### Function enrichment and pathway analysis of the selected module

Next, we used " enrichGO " and " enrichKEGG " functions of the " ClusterProfiler " package to perform GO (Gene Ontology) and KEGG (Kyoto Encyclopedia of Genes and Genomes) enrichment analysis of the “green-yellow” module. Under the conditions of p.adj < 0.05, q value < 0.05, and count ≥ 2, biological processes, cellular components, and molecular functions in GO analysis and signaling pathways in KEGG analysis were identified. The results of the enrichment analysis are presented via a bubble chart.

### Differential expression and survival analysis of the hub genes

Under the condition of Gene Significance(GS) > 0.8 and Module Membership(MM) > 0. 8, the hub genes were screened from the studied modules. Next, the GEPIA database [12] was used to perform a survival analysis of the hub genes. The Kaplan-Meier Plotter database (https://kmplot.com/analysis/) and GSE72094 datasets were used to validate the survival analysis of the hub genes. Furthermore, to confirm result reliability, sequencing data of normal lung tissues from the Genotype-Tissue Expression (GTEx) database (https://commonfund.nih.gov/GTEx) were used with the TCGA LUAD dataset. Next, the TCGA + GTEx, GSE75037, and GSE32863 datasets were selected to verify the differential expressions of the hub genes. Additionally, we downloaded pan-cancer transcriptome data and clinical information from the University of California Santa Cruz (UCSC) Xena browser (https://xena.ucsc.edu/), which included 11,123 samples of 33 cancer types and normal tissues. We extracted the expression data of COL11A1 for each sample. We used R software to calculate the expression differences between normal and tumor samples for each tumor using the Wilcoxon rank-sum test. To explore whether the mRNA levels of the hub genes exhibited diagnostic efficiency for distinguishing LUAD from normal lung tissues, we conducted a receiver operating characteristic (ROC) curve analysis. The pROC package [13] was used to plot ROC curves and calculate the area under the curve (AUC) values in R.

### COL11A1 genomic alteration and promoter methylation in LUAD

C-BioPortal [14] (http://cbioportal.org) is an open-access resource for exploring, visualizing, and analyzing multidimensional cancer genome data. Currently, 225 studies have been conducted on cancer. c-BioPortal was used to analyze changes in COl11A1 gene mutations in the TCGA LUAD samples. UALCAN [15] (http://ualcan.path.uab.edu/) is a comprehensive, user-friendly, and interactive web resource for analyzing cancer OMICS data. UALCAN was used to evaluate the promoter methylation of COL11A1.

### Single‑cell analysis for the expression source of COL11A1

To explore which cells were the main source of COL11A1 gene expression in LUAD, we performed a single-cell analysis using the TISCH database (http://tisch.comp-genomics.org/home/) [16]. TISCH is an scRNA-seq database focusing on the TME and provides detailed cell-type annotation at the single-cell level, enabling the exploration of the TME across different cancer types, including LUAD.

### Immunohistochemistry (IHC) validation of COL11A1

To evaluate the differences in COL11A1 expression at the protein level, paraffin specimens from 30 patients with LUAD were collected from the Pathology Department of the Sixth Affiliated Hospital, Sun Yat-sen University. Each sample contained paired tumors and adjacent normal tissues. This study was approved by the Ethics Committee of the Sixth Affiliated Hospital, Sun Yat-sen University. Written informed consent was obtained from all the patients. IHC was used to assess the expression of COL11A1 in LUAD and adjacent normal tissues. COL11A1 antibody (Proteintech 21841-1-AP-50UL) was used for IHC. Five event horizons were selected randomly and recorded. In this study, ImageJ software and GraphPad Prism 8 were used for quantitative and comparative analyses of the 30 paired samples. Besides, we used immunofluorescence technology to investigate the relationship between COL11A1 and CAFs. We labeled CAFs with a-SMA antibody (Affinity Biosciences AF1032-50).

### Correlation between COL11A1 and immune checkpoint (ICP) genes in human cancers

According to a previous study [17], we collected 60 ICP genes, including 36 immune stimulators and 24 immune inhibitors. Using SangerBox tools, we analyzed the correlation between COL11A1 expression and ICP genes in pan-cancer. Meanwhile, we collected 30 LUAD samples from the sixth affiliated hospital of Sun Yat-sen University, and used RT-qPCR (TB Green® Premix Ex Taq(TaKaRa)) to detect the expression of COL11A1, PD-L1, and CTLA4. β-actin was used as an endogenous control. The primers used in this study were shown as followed:

COL11A1: Forward Primer ACCCTCGCATTGACCTTCC; Reverse Primer TTTGTGCAAAATCCCGTTGTTT;

CD274(PD-L1): Forward Primer TGGCATTTGCTGAACGCATTT; Reverse Primer TGCAGCCAGGTCTAATTGTTTT;

CTLA4: Forward Primer CATGATGGGGAATGAGTTGACC; Reverse Primer TCAGTCCTTGGATAGTGAGGTTC;

β-actin: Forward Primer TGGCACCCAGCACAATGAA; Reverse Primer CTAAGTCATAGTCCGCCTAG;

We set the median expression of COL11A1 as the cut-off point, and divided the 30 LUAD samples into two groups (COL11A1-high and COL11A1-low), and then calculated the differential expression of PD-L1 and CTLA4 between the two groups.

### Statistical analysis

The R version (version 4.1.0) was used for the statistical analysis. Survival analysis was performed according to the Kaplan–Meier analysis and log-rank test. Comparisons of continuous variables were performed using the Student’s t-test or the Wilcoxon rank-sum test, as appropriate. Categorical clinicopathological variables were compared using the Chi-squared test or Fisher’s exact test. Correlation analysis was performed using Spearman’s correlation analysis. A p-value of less than 0.05 was considered statistically significant (ns, p > = 0.05; *, *p* < 0.05; **, *p* < 0.01; ***, *p* < 0.001; ****, *p* < 0.0001).

## Results

### Screening the differentially expressed genes

**Fig. 1:**
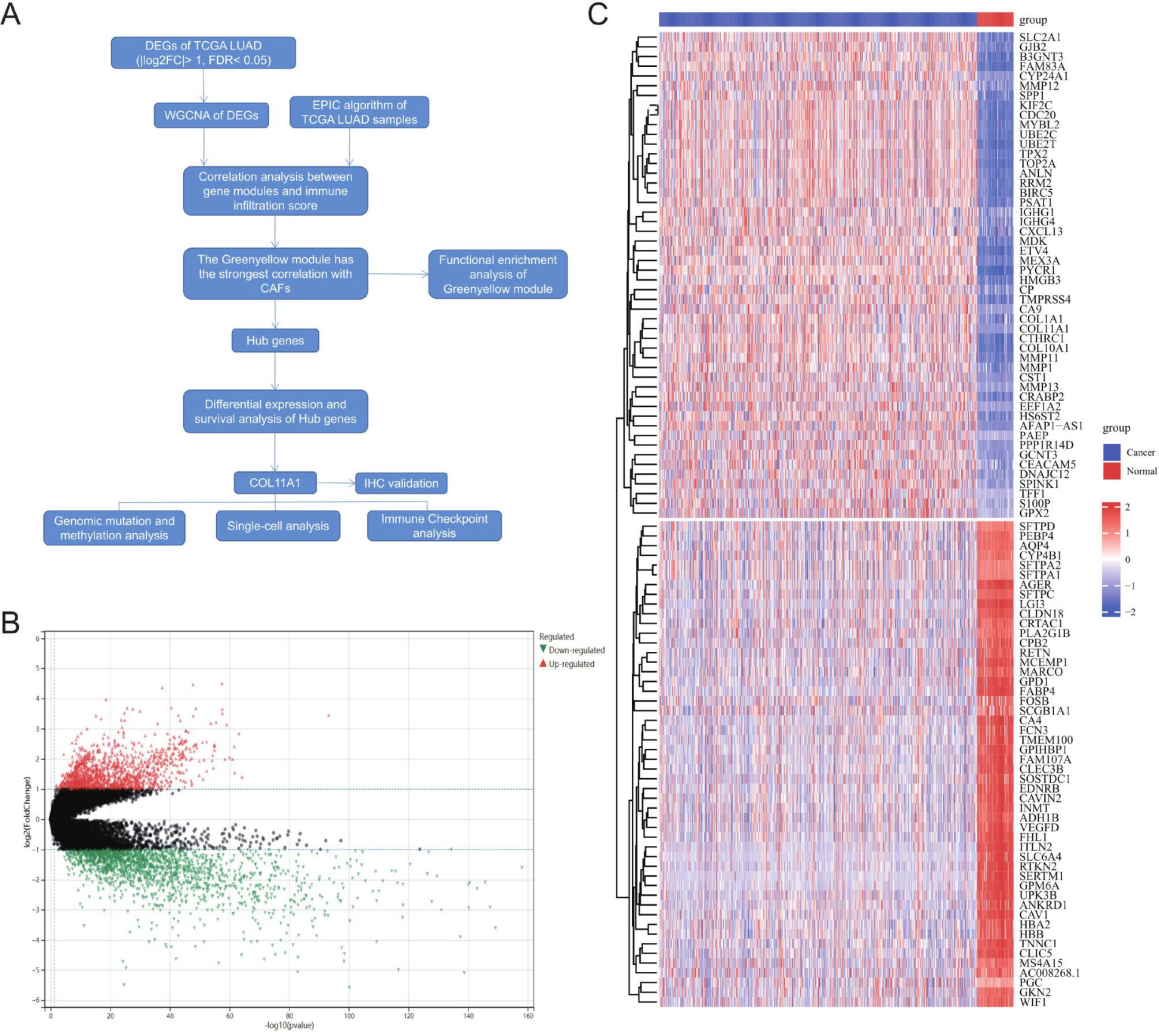
Flow diagram of the data analyzing process and DEGs identification. Identification of differentially expressed genes (DEGs) based on the TCGA LUAD dataset (**A**). Volcano depicts the 3305 DEGs(|log2 FC| >1, FDR < 0.05)in the TCGA LUAD tissues versus normal lung samples **(B)**. Heatmap depicts the top 50 upregulated and top 50 downregulated DEGs **(C)**

The entire data analysis process is described in Fig. 1A. Through screening for differences between tumor and normal tissues in the TCGA LUAD dataset, there were 1412 upregulated and 1893 downregulated genes (|log2FC| >1, FDR < 0.05) (Fig. 1B); The heat map showed the top 50 upregulated and top 50 downregulated DEGs (Fig. 1C).

### Immune infiltration analysis

**Fig. 2:**
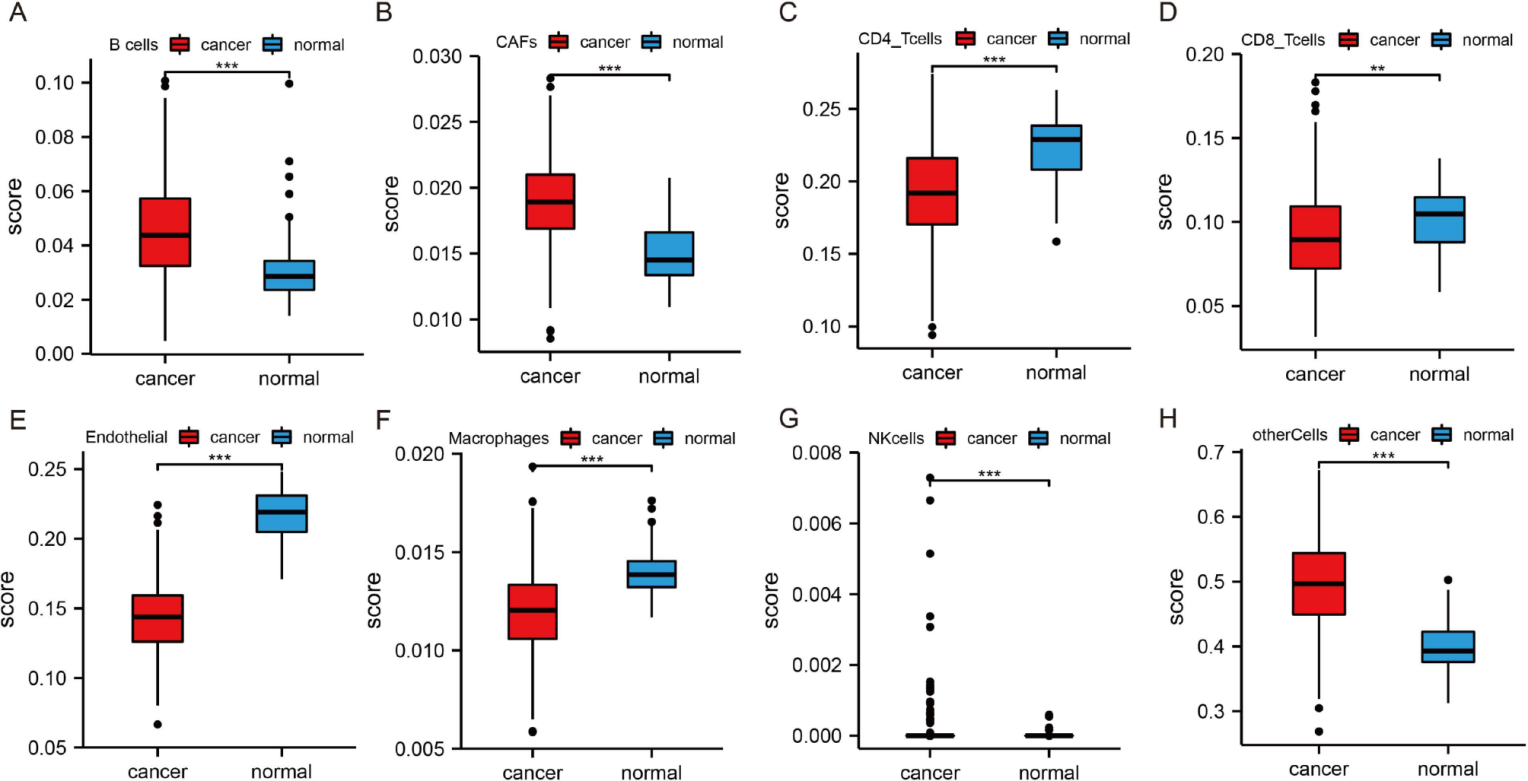
The results from the variance analysis of immune infiltration scores by EPIC algorithm in LUAD B cells (**A**); CAFs (**B**); CD4_T cells (**C**); CD8_T cells (**D**); endothelial (**E**); macrophages (**F**); NK cells (**G**); and other cells (**H**) (**, *p* < 0.01; ***, *p* < 0.001)

To explore the TME infiltration of immune cells, we used the EPIC algorithm (http://epic.gfellerlab.org) to score the immune infiltration for each sample in the TCGA LUAD dataset. The results showed that there were more B cells, CAFs cells, NK cells, and other unclassified cells in LUAD, but fewer CD4 + T cells, CD8 + T cells, macrophages, and endothelial cells**(**Fig. 2A and H**)**. This indicates that CAFs in the TME may have an impact on the growth and development of LUAD.

### WGCNA of DEGs

**Fig. 3:**
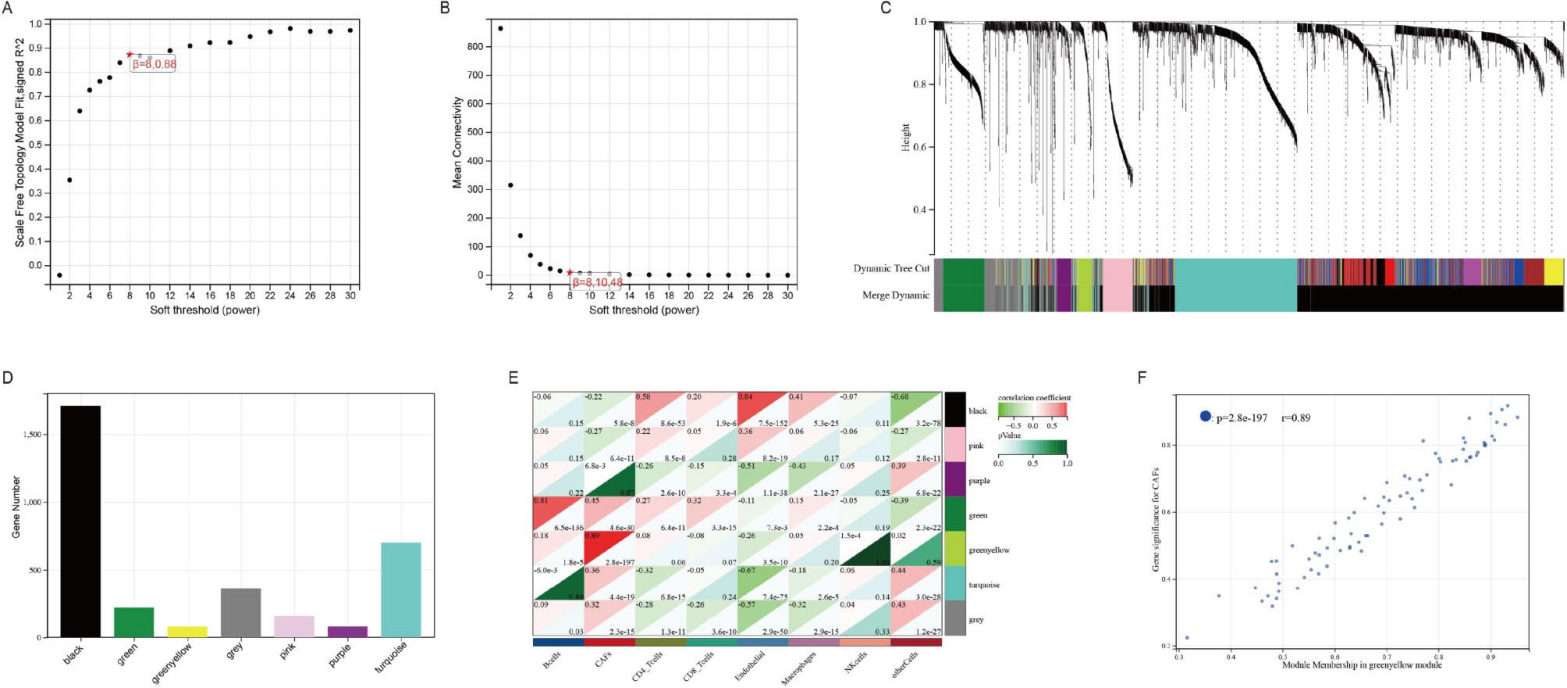
The weighted correlation network analysis of the differentially expressed genes (DEGs). Selection of the optimal soft-thresholding power for the scale-free network and mean connectivity (**A, B**). Dendrogram of 3305 DEGs depending on the dissimilarity measure(1-TOM). Each branch represents a gene, and each color represents a co-expression module (**C**). The number of module genes in the seven modules (**D**). Correlation analysis of the module eigengenes and immune infiltration score by the EPIC algorithm. Spearson’s correlation coefficient and the corresponding p-value are shown (**E**). Scatter plots between the gene significance for CAFs and module membership in the green-yellow module (**F**)

The “WGCNA” package in the R (version 4.1.0) was used to analyze the TCGA LUAD gene expression matrix. Sample clustering analysis was performed on the data to confirm accuracy. After the outlier samples (TCGA-64-5775-01, TCGA-78-7155-01, TCGA-50-6591-01, and TCGA-91-6847-01) were excluded, a sample clustering tree was constructed (Supplementary Fig. 1). To construct a scale-free network, the soft threshold power β was set to 8, independence was set to 0.88, and mean connectivity was close to 0 **(**Fig. 3A and B**)**. DEGs with similar expression patterns were clustered into the same modules, and similar modules were further merged according to the cutting height < 0.25 **(**Fig. 3C**)**. In this study, seven co-expression modules were identified in the TCGA-LUAD dataset. These were black, pink, purple, green, gray, green-yellow, and blue-green modules, respectively (the gray module is composed of genes that cannot be classified) **(**Fig. 3D, Supplementary Table 1). Next, we performed a correlation analysis between the eigenvector of the modules and the immune infiltration score of the samples. Subsequently, we drew the correlation heat map according to the analysis results. We found that the green–yellow module had the strongest correlation with CAFs (*r* = 0.89, *P* = 2.8e-197) **(**Fig. 3E and F**)**. Thus, we selected the green–yellow module to be the important module for further analyses.

### Functional enrichment analyses of the green-yellow module

**Fig. 4:**
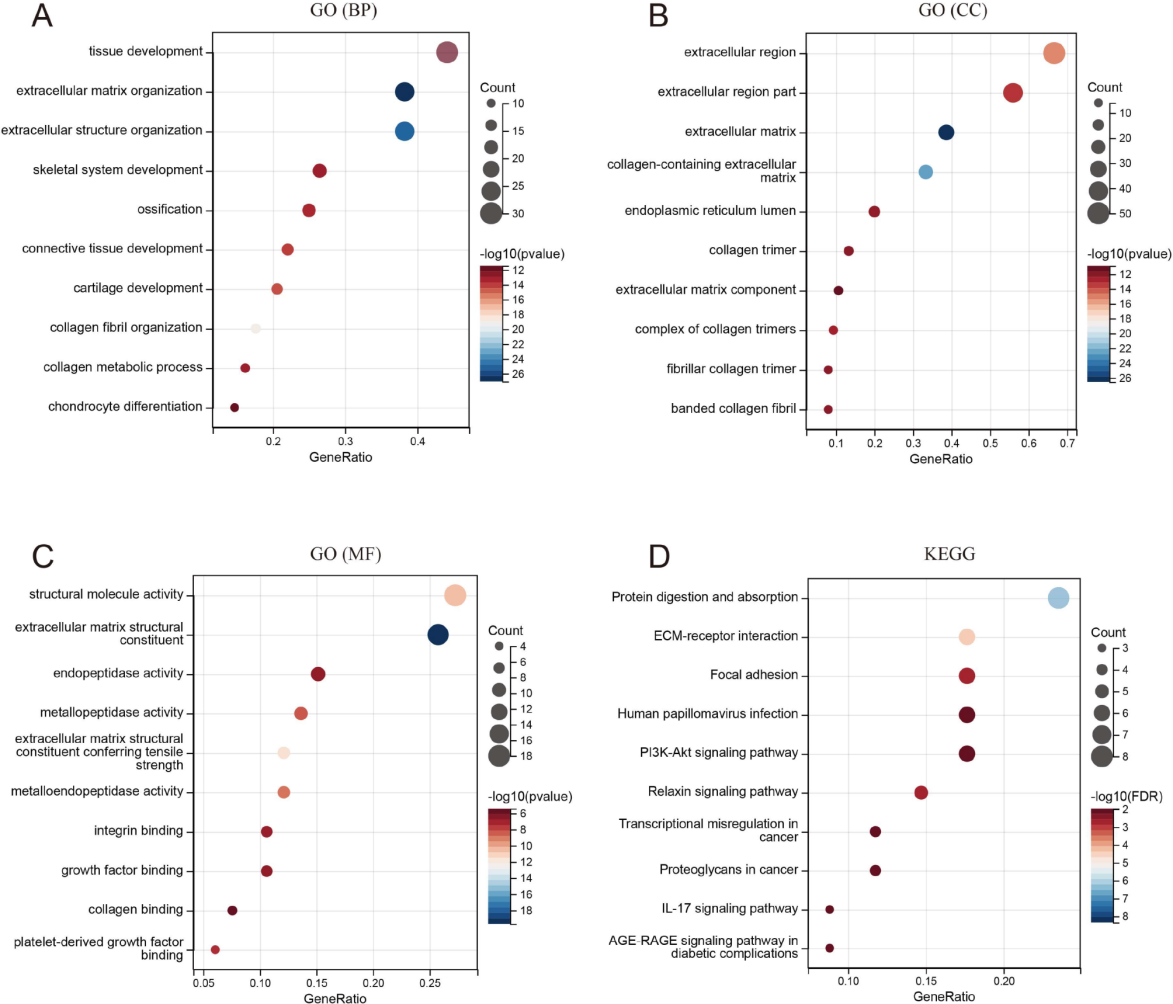
Functional enrichment analyses of the green-yellow module genes. GO functional enrichment analyses of green-yellow module genes (**A–C**). The KEGG functional enrichment analysis of green-yellow module genes (**D**)

The “enrichGO” and “enrichKEGG” functions of the “clusterprofiler” package in Bioconductor were used to perform the GO and KEGG enrichment analysis of the green-yellow module genes. Under the condition of p.adj < 0.05, q value < 0.05 and count ≥ 2, the green-yellow module genes were involved in 256 biological processes (GO-BP), 23 cell components (GO-CC), 38 molecular functions (GO-MF), and 11 KEGG pathways. The bubble graph shows the top 10 messages for GO-BP, GO-CC, GO-MF, and KEGG (Fig. 4A and D). GO functional annotations showed that the green–yellow module genes were mainly involved in tissue development (BP), extracellular matrix organization (BP), extracellular region (CC), extracellular matrix (CC), structural molecule activity (MF), and extracellular matrix structural constituent (MF). The KEGG pathway analysis demonstrated that the green–yellow module genes were primarily associated with protein digestion and absorption, ECM-receptor interaction, focal adhesion, etc. These enrichment analysis results further suggested that the studied module genes were closely related to the TME extracellular matrix.

### Survival analysis of the hub genes and differential expression analysis of COL11A1

**Fig. 5:**
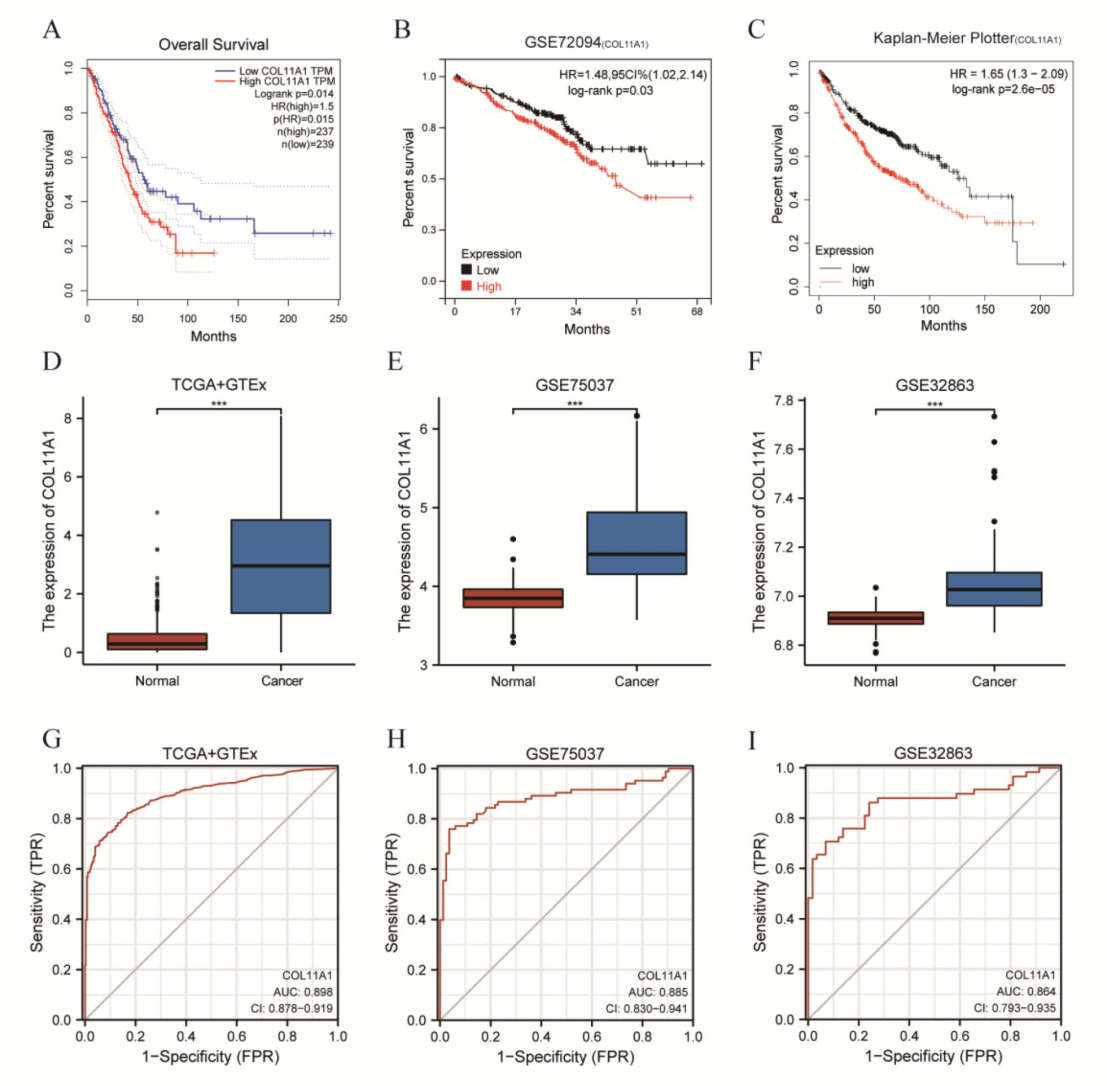
Survival and differential expression analyses of COL11A1. Survival analysis of COL11A1 using the TCGA LUAD dataset (**A**), GSE72094 (**B**), and Kaplan Meier Plotter database (**C**). Differential expression validation of COL11A1 by using TCGA + GTEx, GSE75037, GSE32863 datasets (**D–F**). ROC curve analysis results using TCGA + GTEx, GSE75037, and GSE32863 datasets (**G–I**) (***, *p* < 0.001)

GS > 0.8 and MM > 0.8 were selected as screening conditions, and 13 hub genes related to CAFs were obtained from the green-yellow module. They were ADAM12, ADAMTS12, COL11A1, COL1A1, COL1A2, COL3A1, COL5A1, COL5A2, ITGA11, LRRC15, POSTN, THBS2, and THY1. The GEPIA database was used to analyze the prognosis of the 13 hub genes in LUAD. Taking the median value of each gene expression as the cutoff point, we found that patients with a high COL11A1 expression had a poor prognosis (HR = 1.5, *P* = 0.014) **(**Fig. 5A**)**. Conversely, the other 12 hub genes showed no significant difference in the prognosis of LUAD (Supplementary Fig. 2 We further verified the prognosis of COL11A1 in LUAD using the GSE72094 dataset and Kaplan Meier plotter database and found that patients with LUAD and a high expression of COL11A1 had a poor prognosis (*p* < 0.05) (Fig. 5B and C). These results suggested that patients with a high COL11A1 expression are associated with poor prognosis in LUAD.

**Fig. 6:**
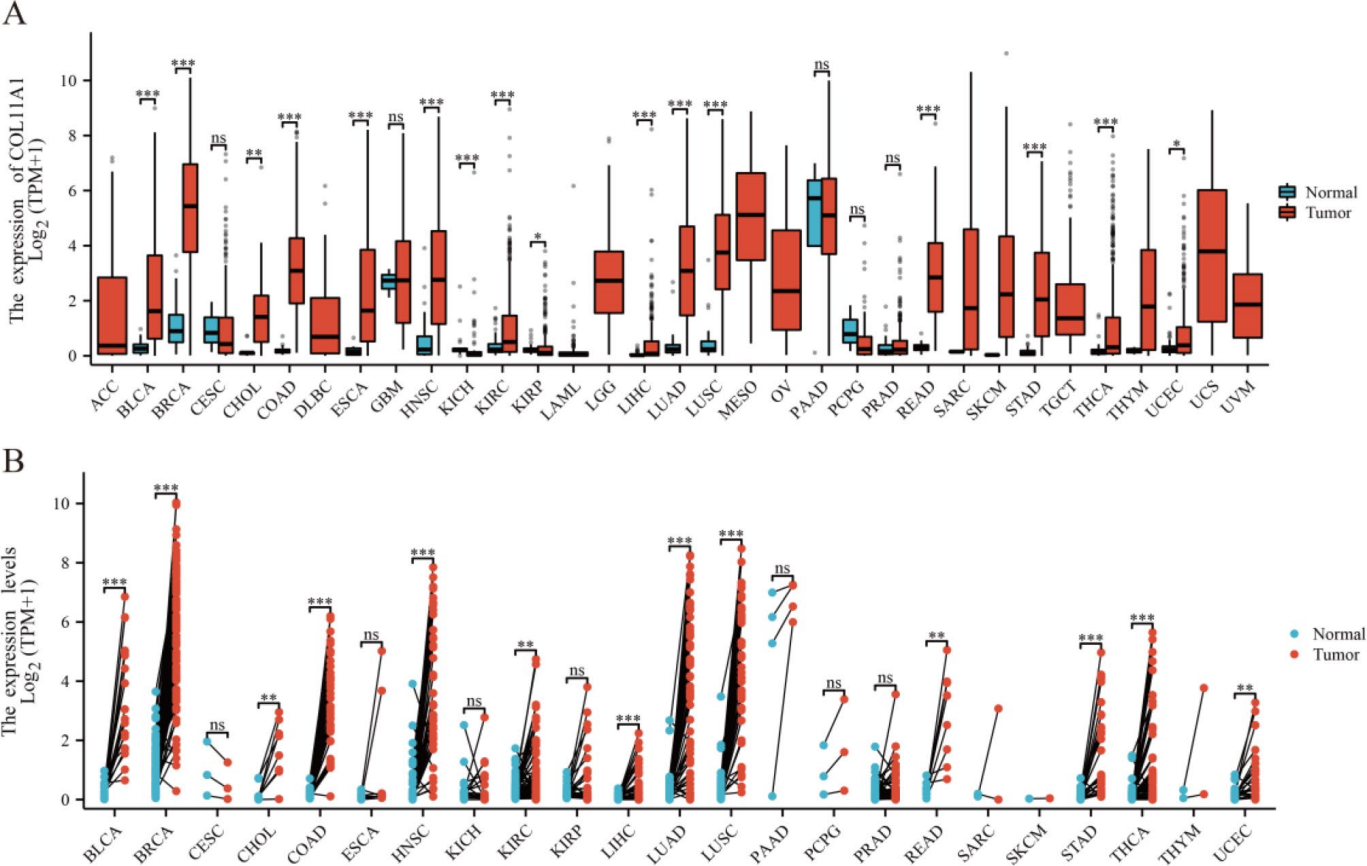
Differential expression analysis of COL11A1 using the pan-cancer dataset. Differential COL11A1 expression between the TCGA cancers and normal tissues (**A**). COL11A1 expression in TCGA cancers and the adjacent paired normal tissues (**B**). ACC, adrenocortical carcinoma; BLCA, bladder urothelial carcinoma; BRCA, breast invasive carcinoma; CESC, cervical squamous cell carcinoma and endocervical adenocarcinoma; CHOL, cholangiocarcinoma; COAD, colon adenocarcinoma; DLBC, lymphoid neoplasm diffuse large B-cell lymphoma; ESCA, esophageal carcinoma; GBM, glioblastoma multiforme; HNSC, head and neck squamous cell carcinoma; KICH, kidney chromophobe; KIRC, kidney renal clear cell carcinoma; KIRP, kidney renal papillary cell carcinoma; LAML, acute myeloid leukemia; LGG, brain lower grade glioma; LIHC, liver hepatocellular carcinoma; LUAD, lung adenocarcinoma; LUSC, lung squamous cell carcinoma; MESO, mesothelioma; OV, ovarian serous cystadenocarcinoma; PAAD, pancreatic adenocarcinoma; PCPG, pheochromocytoma and paraganglioma; PRAD, prostate adenocarcinoma; READ rectum adenocarcinoma; SARC, sarcoma; SKCM, skin cutaneous melanoma; STAD, stomach adenocarcinoma; TGCT, testicular germ cell tumor; THCA, thyroid carcinoma; THYM, thymoma; UCEC, uterine corpus endometrial carcinoma; UCS, uterine carcinosarcoma; UVM uveal melanoma. (ns, *p* ≥ 0.05; *, *p* < 0.05; **, *p* < 0.01; ***, *p* < 0.001)

Based on the TCGA + GTEx database and GSE32863 and GSE75037 datasets, we further verified that the expression of COL11A1 in LUAD tissues was significantly higher than that in normal lung tissues (*p* < 0.0001) (Fig. 5D and F). Furthermore, the ROC analysis results showed that the AUC values were 0.898 (TCGA + GTEx) (Fig. 5G), 0.885 (GSE75037) (Fig. 5H), and 0.864 (GSE32863) (Fig. 5I), respectively. To further understand the expression of COL11A1 in cancers, we also evaluated COL11A1 expression in TCGA pan-cancer dataset. The results showed that COL11A1 expression was significantly upregulated in 15 cancer types (BLCA, BRCA, CHOL, COAD, ESCA, HNSC, KIRC, KIRP, LIHC, LUAD, LUSC, READ, STAD, THCA, and UCEC) **(**Fig. 6A**)**. For paired tumors and normal tissues in the TCGA pan-cancer dataset, COL11A1 expression was significantly higher in BLCA, BRCA, CHOL, COAD, ESCA, HNSC, KIRC, LIHC, LUAD, LUSC, READ, STAD, THCA, and UCEC **(**Fig. 6B**)**. These results suggested that COL11A1 may play an important role in the proliferation of cancers.

### Genetic alteration and methylation analysis of COL11A1 in LUAD

**Fig. 7:**
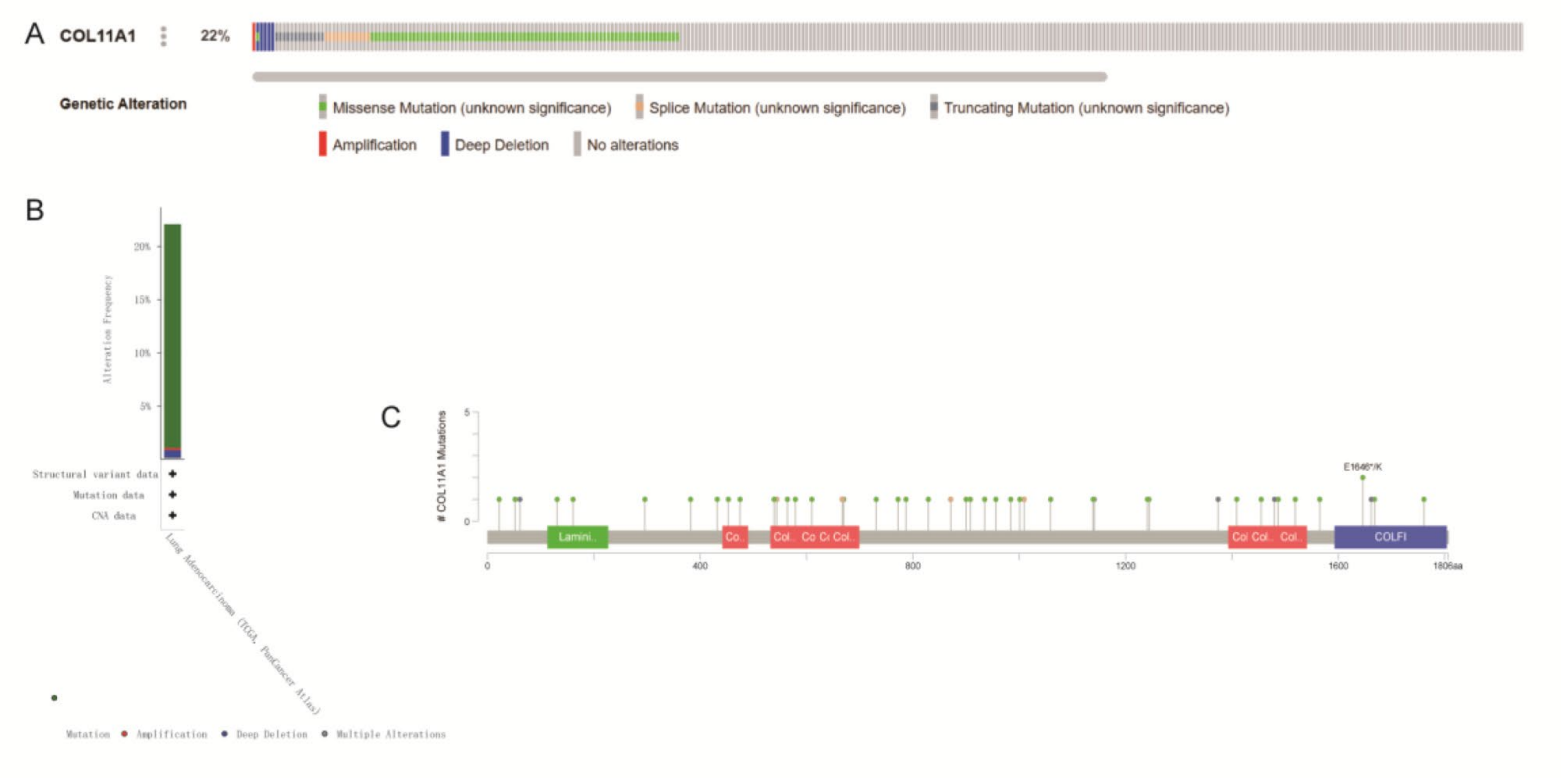
COL11A1 genomic alterations and methylation were analyzed using the cBioPortal database and ULACAN database. OncoPrint of COL11A1 gene alterations in the TCGA LUAD cohort (different colors = different types of genetic alterations) (**A**). COL11A1 alteration types and the frequency of TCGA LUAD (**B**). Mutation sites of the COL11A1 gene in TCGA LUAD (**C**)

The above results indicate that when COL11A1 is highly expressed in LUAD, the prognosis is poor. Genetic alterations are one of the key factors affecting gene expression [18]. We used the TCGA LUAD dataset from the cBioPortal database to explore the genetic alterations of COL11A1. In total, 584 patients were included in this study. A total of 22% of the patients had gene mutations, including missense mutations, splice mutations, truncating mutations, amplification, and deep deletion **(**Fig. 7A and B**)**. A total of 46 mutation sites were found in patients with these mutations **(**Fig. 7C**)**. These findings indicated that COL11A1 has a relatively high frequency of genetic alterations in LUAD.

Epigenetic modifications, such as DNA promoter methylation regulate gene expression, and affect the growth and development of cancers [19]. Therefore, to explore the cause for a high expression of COL11A1 in LUAD, we analyzed DNA promoter methylation. We used the UALCAN database to explore COL11A1 methylation in patients with TCGA LUAD. The results showed that the methylation level of COL11A1 in tumor tissues is significantly higher than that in normal tissues (*p* = 1.62e-12) (Supplementary Fig. 3).

### Single-cell analysis of COL11A1

**Fig. 8:**
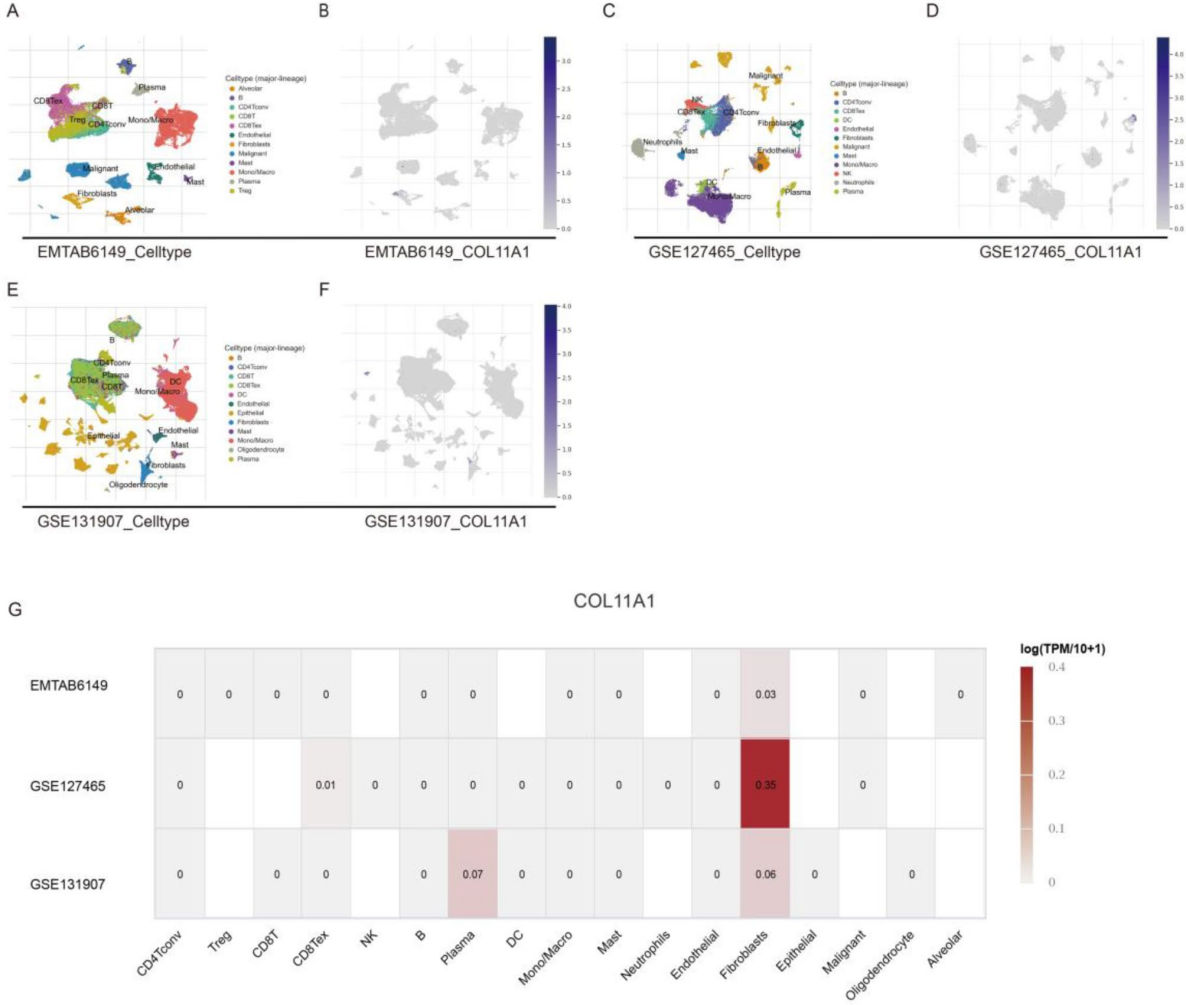
The expression source of COL11A1 is shown by single-cell analysis. UMAP plot of EMTAB6149 (**A**, **B**). UMAP plot of GSE127465 (**C**, **D**). UMAP plot of GSE131907 (**E**, **F**). The expression level of COL11A1 in the different cell types of the EMTAB6149, GSE127465, and GSE131907 datasets (**G**)

Single-cell analysis can provide a profound understanding of the biological characteristics of cancers. Three LUAD datasets from the TISCH database were used for the analysis, namely, the EMTAB6149, GSE127465, and GSE131907 datasets. The UMAP algorithm was used for dimensionality reduction and visualization of the three datasets. In the EMTAB6149 dataset, we observed that COL11A1 was mainly expressed in CAFs **(**Fig. 8A and B**)**. In the GSE127465 dataset, we observed that COL11A1 was mainly expressed in CAFs and CD8 tex cells **(**Fig. 8C and D**)**. In the GSE131907 dataset, we observed that COL11A1 was mainly expressed in plasma cells and CAFs **(**Fig. 8E and F**)**. The quantitative expression of COL11A1 in the TME of the corresponding datasets is represented in Fig. 8G. These results further confirmed the strong correlation between COL11A1 and CAFs. Collectively, these results suggested that COL11A1 in the TME is mainly produced by CAFs.

### Immunohistochemical validation for the expression of COL11A1 in LUAD

**Fig. 9:**
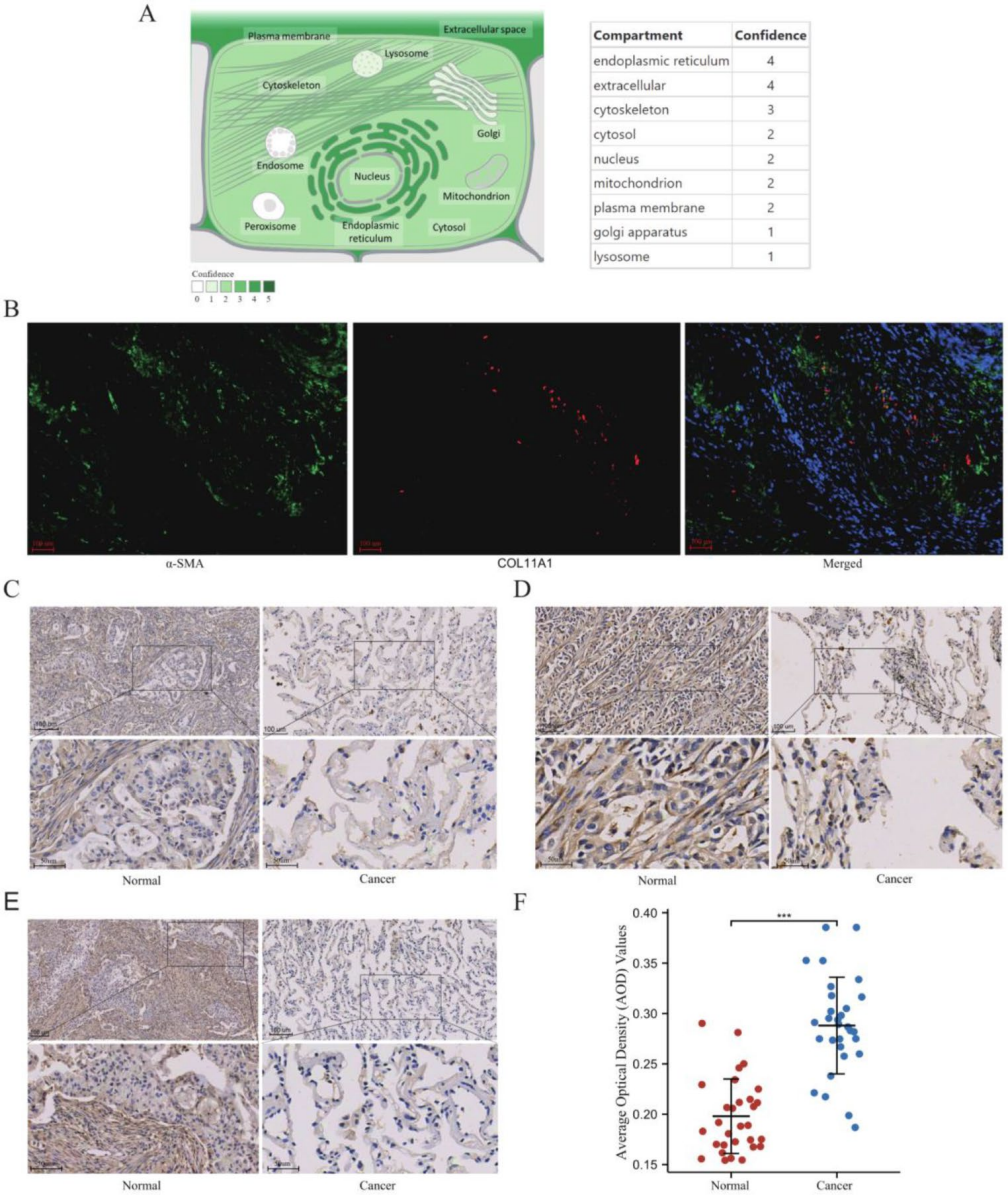
Immunohistochemical validation for the expression of COL11A1 in LUAD Subcellular localization for COL11A1 using the Genecard database (**A**). Typical representatives of the immunofluorescence images of COL11A1 in LUAD samples (**B**). Typical representatives of the immunohistochemical data of COL11A1 in 3 paired LUAD samples (**C**, **D**, **F**). The statistical results of differential expression of COL11A1 in 30 paired LUAD samples (**F**) (***, *p* < 0.001)

Using the GeneCards database, subcellular location information for the COL11A1 gene showed that it is mainly expressed in the endoplasmic reticulum and extracellular space (Fig. 9A**)**. For validation at the experimental level, we analyzed COL11A1 protein expression by IHC staining. Thirty pairs of samples from 30 patients with LUAD in the Sixth Affiliated Hospital, Sun Yat-sen University were collected. Typical images of the three patients are represented in Fig. 9C and E. The results showed that COL11A1 was mainly enriched in the stromal tissues of LUAD, whereas weak staining or even non-staining was observed in normal tissues. Utilizing immunofluorescence method, We found red fluorescence and green fluorescence basically overlap, which supported that COL11A1 was mainly expressed by fibroblasts (Red: COL11A1; Green: α-SMA) (Fig. 9B**)**. By randomly selecting five fields of view from each sample and using Image J to determine the average optical density(AOD) value, we found that the expression of COL11A1 in LUAD tissues was significantly higher than that in normal tissues (*P* < 0.001) (Fig. 9F**).** The above results indicated that COL11A1 is highly expressed in LUAD at the protein level, and associated with CAFs.

### COL11A1 is related to ICP genes in human cancers

**Fig. 10:**
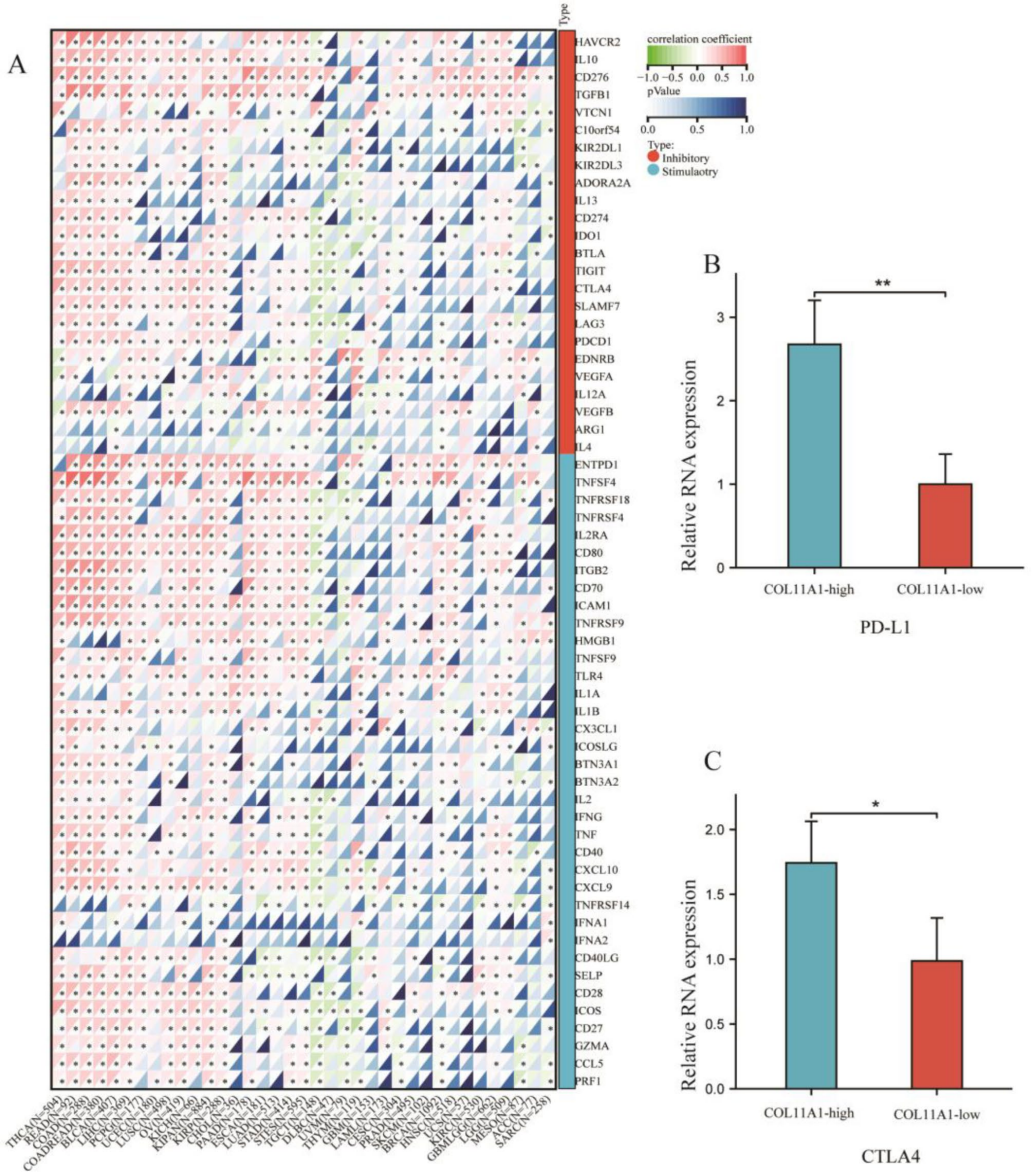
Correlation between COL11A1 and immune checkpoint genes (**A**). PD-L1 and CTLA4 expression in the COL11A1-high group and the COL11A1-low group was measured by qPCR (**B**, **C**) (*, *p* < 0.05; ***, *p* < 0.001)

Immune surveillance affects the growth and development of cancer cells, which evade immune responses by the ICP [20]. We investigated the association between COL11A1 expression and ICP genes in human cancers to explore the potential function of COL11A1 in immunotherapy. The results showed a correlation between COL11A1 and ICP genes in the 37 tumor types. The ICP genes are divided into two major categories: immunoinhibitors and immunostimulators. We found that the expression of COL11A1 is positively correlated with most immunoinhibitors and immunostimulators in thyroid carcinoma, rectal adenocarcinoma, colon adenocarcinoma, colon adenocarcinoma/rectum adenocarcinoma, bladder urothelial carcinoma, liver hepatocellular carcinoma, pheochromocytoma and paraganglioma, uterine corpus endometrial carcinoma, lung squamous cell carcinoma, ovarian serous cystadenocarcinoma, kidney chromophobe, pan-kidney cohort, kidney renal papillary cell carcinoma, pancreatic adenocarcinoma, lung adenocarcinoma, stomach adenocarcinoma, stomach and esophageal carcinoma, glioma, and brain low-grade glioma. The expression of COL11A1 is negatively correlated with most immunoinhibitors and immunostimulators in testicular germ cell tumors and uveal melanoma. Notably, COL11A1 positively correlated with HAVCR2(TIM3), CD274 (PD-L1), CTLA4, and LAG3 in LUAD(Fig. 10A). Then, we collected 30 LUAD samples and used RT-qPCR to detect the expression of COL11A1, PD-L1, and CTLA4. We found that the expression of PD-1 and CTLA4 in the COL11A1-high group was much higher than that in the COL11A1-low group (Fig. 10B and C) (all, *p* < 0.05). These findings suggested that COL11A1 may influence the regulation of TME in human cancers.

## Discussion

Although LUAD has made good progress in diagnosis and treatment, it has a high degree of malignancy and a poor survival prognosis. Exploring the detailed mechanisms of LUAD pathogenesis and identifying promising biomarkers for LUAD may help provide effective therapeutic targets and improve patient outcomes [21, 22]. Many studies have suggested that CAFs in the tumor microenvironment are important factors affecting tumor invasion and metastasis [23]. Therefore, we aimed to screen for biomarkers that affect the prognosis and diagnosis of LUAD from the perspective of CAFs in the TME. The results of our study comprehensively support the idea that COL11A1 derived from CAFs in the TME of LUAD may serve as a novel prognostic and therapeutic biomarker for LUAD.

The WGCNA and EPIC algorithms for immune infiltration were used to analyze the LUAD sample data in TCGA, and 13 hub genes with a strong correlation with CAFs were obtained. They were ADAM12, ADAMTS12, COL11A1, COL1A1, COL1A2, COL3A1, COL5A1, COL5A2, ITGA11, LRRC15, POSTN, THBS2, THY1. To clarify the prognosis of the 13 hub genes in LUAD, the GEPIA database was used for analysis, and it was found that the COL11A1 gene was highly expressed in LUAD and had a poor prognosis. We further verified the effect of COL11A1 on the prognosis of LUAD patients using the GSE72094 and Kaplan-Meier plotter databases and confirmed that the prognosis of LUAD patients with high expression of COL11A1 was poor. Feng et al. demonstrated that COL11A1 is a potential effector gene that is positively regulated by MPZL1 and correlates with poor prognosis in LUAD patients [24]. Therefore, COL11A1 may be a potential prognostic biomarker in LUAD.

To date, 28 collagen types with different structures and biological functions [25]. Increasing evidence has emphasized the role of collagen in promoting cancer cell proliferation and creating a tumor microenvironment for metastasis [26]. Among the 13 hub genes, 6 belonged to the collagen family. These are COL11A1, COL1A1, COL1A2, COL3A1, COL5A1, and COL5A2, respectively. Although five genes(COL1A1, COL1A2, COL3A1, COL5A1, and COL5A2) did not show significance on the prognosis of LUAD patients in our study, previous studies have reported that these genes would affect tumor growth. Liu et al. discovered that COL1A1 promotes the metastasis of breast cancer and is a potential therapeutic target [27]. Wang et al. demonstrated that COL1A2 inhibition attenuated GBM proliferation by promoting cell cycle arrest [28]. Shen et al. revealed that COL3A1 expression is an independent prognostic predictor in HNSCC patients [29]. Yang et al. found that COL5A1 promotes the progression of gastric cancer by acting as a ceRNA of miR-137-3p to upregulate FSTL1 expression [30]. Ren et al. indicated that COL5A2 can promote cell proliferation and invasion in Prostate Cancer [31]. Therefore, collagen plays an important role in tumorigenesis and development. Currently, most studies on collagen have focused on individual subtypes, and further exploration is needed on the interactions among collagen subtypes.

COL11A1 is a component of XI collagen and is found mainly in the cartilage [32]. The lack of type XI collagen leads to abnormal thickening of cartilage tissues in cartilage [33, 34]. Importantly, emerging evidence indicates that COL11A1 is associated with cancer progression that can promote tumor growth, migration, invasion, metastasis, and chemotherapy resistance [35]. In addition, upregulation of COL11A1 is associated with cancer recurrence and poor survival and in some types of cancer, such as breast, colorectal, gastric, and so on [36–39]. Several reports have also validated that COL11A1 is an oncogene in the progression of non-small cell lung cancer [40–43]. However, there are few reports on the effect of COL11A1 on immune infiltration in the TME of LUAD.

In our study, we used TCGA + GTEx, GSE32863, and GSE75037 datasets to confirm that the expression of COL11A1 was significantly higher in tumor tissues than in normal tissues, which was verified by our own samples in IHC experiments. In addition, the corresponding AUC values of the ROC curves were all greater than 0.86, which revealed that COL11A1 exhibited excellent efficiency in distinguishing LUAD from adjacent normal tissues. Chong et al. reported that COL11A1 could be a biomarker for the diagnosis of non-small cell lung cancer [44]. We also found that COL11A1 is overexpressed in many cancer types. Therefore, high expression of COL11A1 alone is not yet used as a diagnostic specific biomarker of LUAD. In the future, further exploration is needed to investigate whether COL11A1 could be a pan-caner biomarker like the carcinoembryonic antigen (CEA) and carbohydrate antigen 19 − 9 (CA-19-9) [45].

To explore the reasons for the high expression of COL11A1 in LUAD, we used the c-BioPortal database to study genetic alterations in COL11A1. We found that the gene mutation rate of COL11A1 in LUAD was as high as 22%. COL11A1 gene mutation may disrupt certain regulatory elements, leading to high expression of COL11A1 in LUAD. Gene promoter methylation is another important factor that affects gene expression. The ULCAN database was used to explore the promoter methylation of COL11A1 in LUAD. We found that the tumor tissues were hypermethylated compared to the adjacent normal tissues, which could not explain why the COL11A1 gene was highly expressed in LUAD. Therefore, it is suggested that promoter methylation is not the cause of the high expression of COL11A1 in LUAD. Of note, in addition to gene mutations and promoter methylation, the expression of COL11A1 at the transcriptional level may also be affected by many other factors, such as histone modification and m6A RNA methylation modification [46–48], which have not been discussed in our study.

To explore the mechanism by which COL11A1 affects the proliferation and invasion of LUAD cells, we performed GO and KEGG analyses. We found that the genes co-expressed with COL11A1 (green–yellow module) were mainly enriched in signal pathways related to the extracellular matrix in the TME of LUAD. Lee et al. revealed that COL11A1 is enriched in β1 integrin signaling, focal adhesion, and features of extracellular matrix-receptor interactions [49]. These results further suggested that COL11A1 plays an indispensable role in influencing tumor proliferation and invasion through the TME. In recent years, single-cell RNA sequencing technology has developed rapidly. This allows us to master information that is not available by bulk RNA sequencing [50]. In our study, using a single-cell analysis-related database, we found that COL11A1 was mainly expressed by CAFs in the TME rather than from LUAD cancer cells, which was also supported by our results of immunofluorescence. Jia et al. revealed that COL11A1 is a highly specific biomarker of activated CAFs [51]. Therefore, we believe that CAFs in the TME may affect the growth and development of LUAD cells by COL11A1.

ICP genes affect immune cell infiltration in the TME and cancer immunotherapy [52]. We analyzed the relationship between COL11A1 and ICP gene expression. The results showed a correlation between the COL11A1 and ICP genes in all 37 tumors. This indicates that COL11A1 affects immune function in the TME. Notably, COL11A1 positively correlated with HAVCR2(TIM3), CD274 (PD-L1), CTLA4, and LAG3 in LUAD. Using RT-qPCR, we found that the expression of PD-1 and CTLA4 in the COL11A1-high group was much higher than that in the COL11A1-low group. PD-L1, TIM3, LAG3, and CTLA4 are essential biomarkers of T cell exhaustion [53]. Therefore, a high expression of COL11A1 may result in T cell exhaustion in the TME of LUAD. T-cell exhaustion is defined as the impairment or even loss of T-cell activities in patients with common chronic infections or cancers. The discovery and clinical application of immune checkpoint inhibitors targeting CTLA4 and PD-L1 have revolutionized cancer therapy [54]. Therefore, the expression of COL11A1 in LUAD may affect the efficacy of cancer immunotherapy.

Our study has some limitations. First, we found a correlation between COL11A1 and T cell exhaustion, but our results were generated mainly through bioinformatic analysis. To date, there is still no research on how COL11A1 results in T cell exhaustion in the TME of LUAD. This aspect will be one of our future research directions. Second, the data was retrospective. In the future, our results should be further confirmed via prospective studies. Third, we found that COL11A1 was overexpressed in LUAD at both the mRNA and protein levels. In the future, to increase the clinical application of COL11A1, we need to collect patients’ blood, pleural fluid, and sputum samples to further explore COL11A1 expression.

## Conclusion

In summary, COL11A1 is mainly expressed and secreted by CAFs. A high expression of COL11A1 may result in T cell exhaustion in the TME of lung adenocarcinoma. COL11A1 may serve as a novel prognostic biomarker and provide new insights into LUAD therapeutics, particularly cancer immunotherapy.

### Electronic supplementary material

Below is the link to the electronic supplementary material.

Supplementary Material 1

Supplementary Material 2

Supplementary Material 3

Supplementary Material 4

## Data Availability

Publicly available datasets were analyzed in this study. This data is derived from the LUAD cohort from the TCGA database (https://portal.gdc.cancer.gov/), the GSE72094, GSE75037, and GSE32863 datasets from the GEO database, (https://www.ncbi.nlm.nih.gov/geo), the pan-cancer cohort from the University of California Santa Cruz (UCSC)’s Xena browser (https://xena.ucsc.edu/), and data of normal lung tissues from the Genotype-Tissue Expression (GTEx) database (https://commonfund.nih.gov/GTEx).The original contributions presented in the study are included in the Supplementary Materials. Further inquiries can be directed to the corresponding author.

## Abbreviations

CAFs: Cancer-associated fibroblasts
DEGs: Differentially expressed genes
GEO: Gene Expression Omnibus
GO: Gene ontology
ICP: Immune checkpoint
IHC: Immunohistochemistry
KEGG: Kyoto Encyclopedia of Genes and Genomes
LUAD: Lung adenocarcinoma
TME: Tumor microenvironment
WGCNA: Weighted gene co-expression network analysis
TCGA: The Cancer Genome Atlas
