# Supplementary figures and images for: Analysis of cancer-associated fibroblasts related genes identifies COL11A1 associated with lung adenocarcinoma prognosis
Source: BMC Med Genomics. 2024 Apr 22;17:97. doi: 10.1186/s12920-024-01863-1 (PMC11036680; doi:10.1186/s12920-024-01863-1)

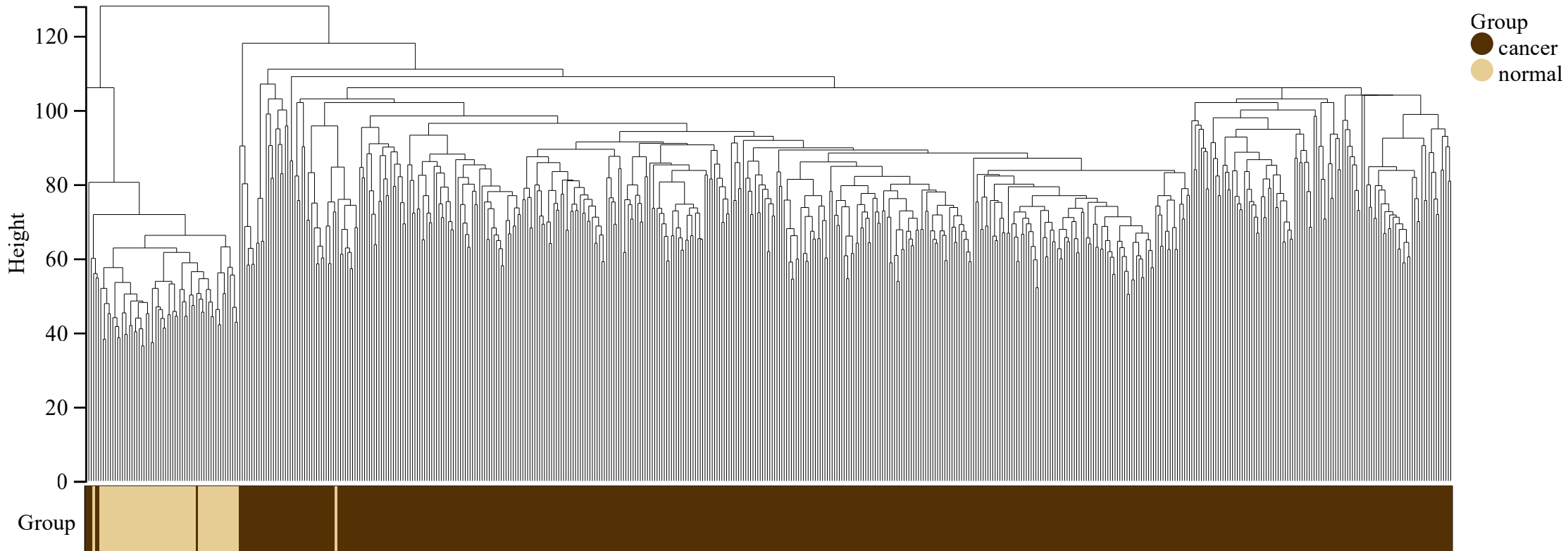

Supplement: Supplementary file 2 — Supplementary Material 2 [file 12920_2024_1863_MOESM2_ESM.pdf]

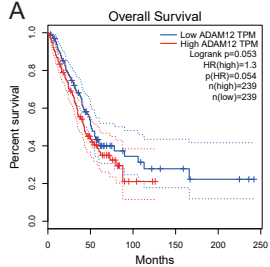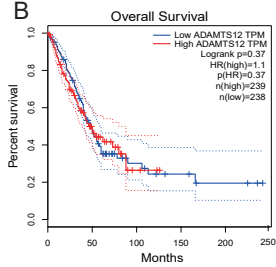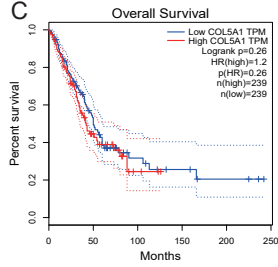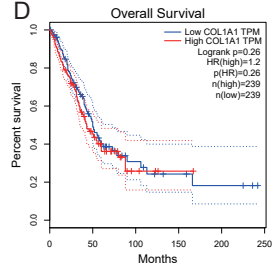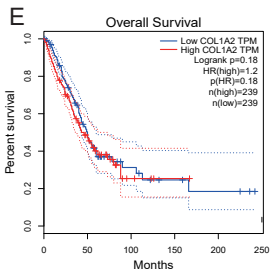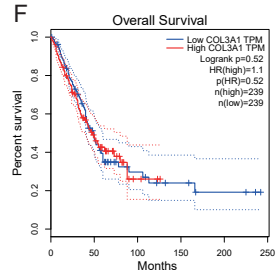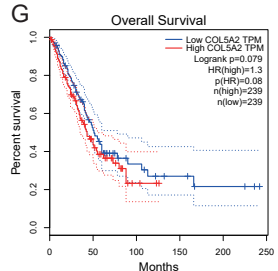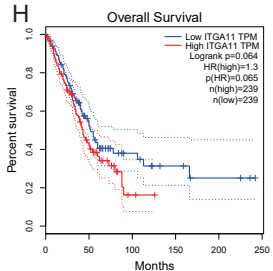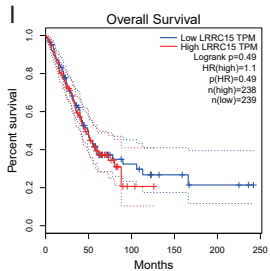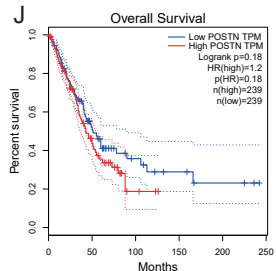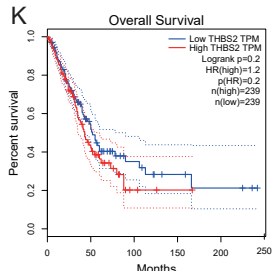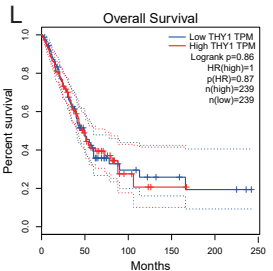

Supplement: Supplementary file 3 — Supplementary Material 3 [file 12920_2024_1863_MOESM3_ESM.pdf]

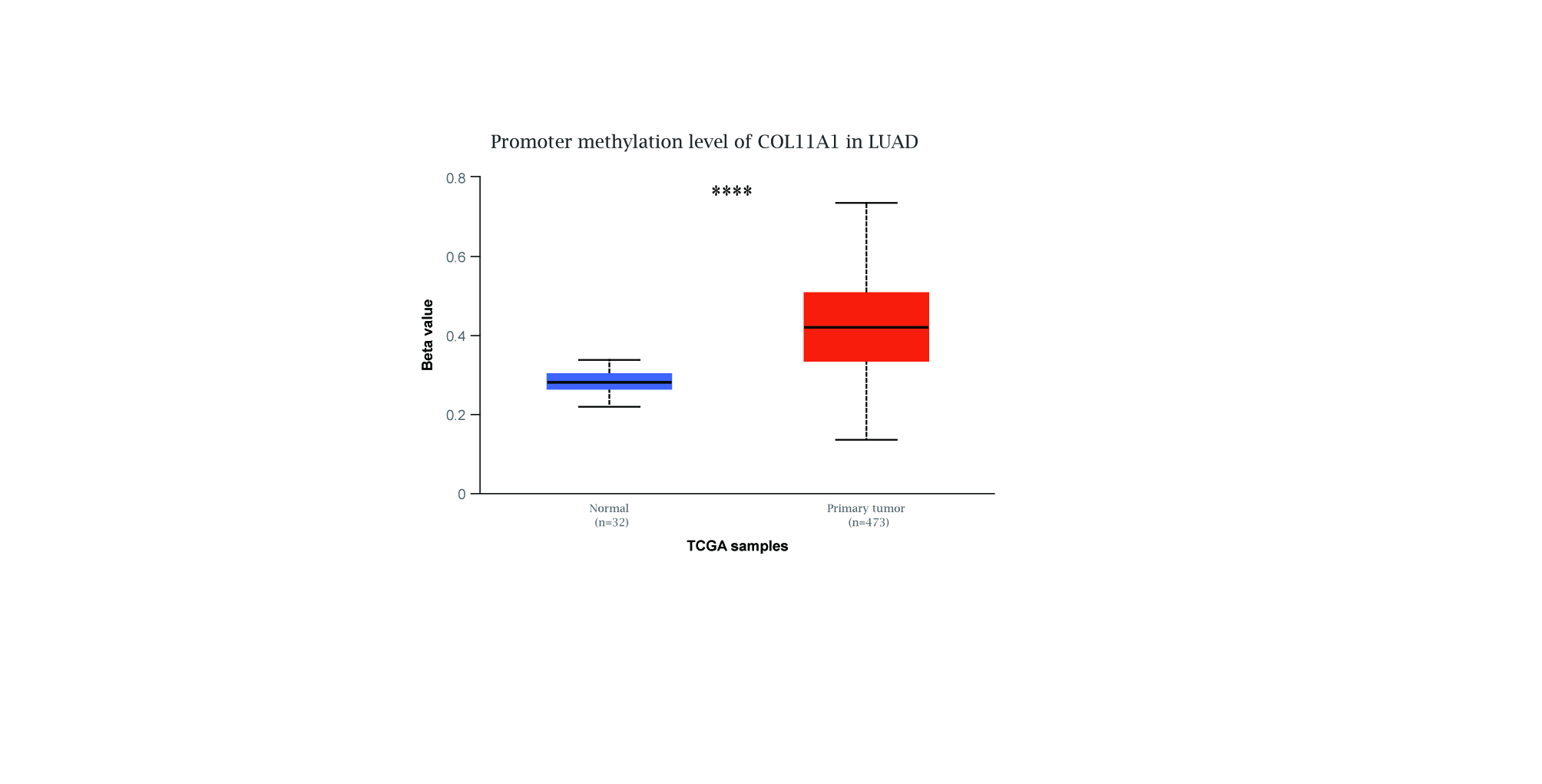

Supplement: Supplementary file 4 — Supplementary Material 4 [file 12920_2024_1863_MOESM4_ESM.tif]
